# Homing in on an intracellular target for delivery of loaded nanoparticles functionalized with a histone deacetylase inhibitor

**DOI:** 10.18632/oncotarget.20021

**Published:** 2017-08-07

**Authors:** Jie Zhang, Yaling Shi, Yueqin Zheng, Chengcheng Pan, Xiaoying Yang, Taoyan Dou, Binghe Wang, Wen Lu

**Affiliations:** ^1^ School of Pharmacy, Health Science Center, Xi'an Jiaotong University, Xi’an, 710061, P.R. China; ^2^ Department of Chemistry and Center for Diagnostics and Therapeutics, Georgia State University, Atlanta, Georgia 30303, USA

**Keywords:** drug delivery, histone deacetylase inhibitor, intracellular targeting, functionalized PLGA, cellular uptake

## Abstract

Functionalized nanoparticles (NPs) are usually used to enhance cellular penetration for targeted drug delivery that can improve efficacy and reduce side effects. However, it is difficult to exploit intracellular targets for similar delivery applications. Herein we describe the targeted delivery of functionalized NPs by homing in on an intracellular target, histone deacetylases (HDACs). Specifically, a modified poly-lactide-co-glycolideacid (FPLGA) was yielded by conjugation with an HDAC inhibitor. Subsequently, FPLGA was used to prepare functionalized FPLGA NPs. Compared to unmodified NPs, FPLGA NPs were more efficiently uptaken or retained by MCF-7 cells and showed longer retention time intracellular. *In vivo* fluorescence imaging also revealed that they had a higher accumulation and a slower elimination than unmodified NPs. FPLGA NPs loaded with paclitaxel exhibited superior anticancer efficacy compared with unmodified NPs. These results offer a promising approach for intracellular drug delivery through elevating the concentration of NPs.

## INTRODUCTION

The reliance on systemic administration of traditional cytotoxic agents in cancer treatment has been the root of some severe side effects in cancer therapy. Therefore, there has been a strong interest in developing therapies aimed at molecular targets unique in cancer or finding ways to target cancer in the delivery of cytotoxic agents. Along this line, drugs such as Gleevec demonstrated the tremendous potential in inhibiting a unique cancer target, the Bcr-Abl tyrosine kinase. Corollary to this is the pursuit of ways to target cytotoxic agents to cancer with enhanced concentrations. Such approaches include antibody-drug conjugates [1], nanoparticle-based delivery [2], and small molecule-drug conjugates [3] among others [4], which take advantage of the specific recognition of cell surface biomarkers for targeted delivery. Homing in on an intracellular target has been challenging for obvious reasons. Histone deacetylases (HDACs) play an essential role in various biological processes, including the modulation of chromatin topology, gene transcription and epigenetic modulation. Epigenetic modifications are critical to the deregulation of gene expression during cancer development. As a result, HDACs play a vital role in cell proliferation, cell-cycle regulation and apoptosis [5], and elevated expression of HDACs has been observed in many cancer types including breast, stomach, esophageal, colon, prostate, ovarian, lung and pancreatic cancer [6, 7]. Therefore, HDACs have been considered as promising targets for the treatment of cancer. In this manuscript, we examine the feasibility of targeting HDACs for enhanced intracellular delivery. The basic premise of this idea is that elevated expression levels of HDACs can be used to “trap” in place those molecules that can bind to HDACs. For this purpose, we were interested in examining the utility of a nanoparticle vehicle for targeted delivery of cytotoxic agents, such as paclitaxel, based on the enhanced concentration of HDACs.

The application of nanotechnology in cancer therapy is widespread, particularly in targeted drug delivery systems [8]. The first generation of nanoparticles were designed to passively target and accumulate in tumors through the enhanced permeability and retention (EPR) effect due to porous vasculature and thus enhanced permeability in malignant tumor [9]. However, uptake by macrophages in the reticuloendothelial system often limits their therapeutic efficacy [10] and leads to side effects. In recent years, active targeting approaches have been exploited to improve the accumulation of nanoparticles in particular organs, tissues and cells. Active targeting relies on the use of specific ligands that recognize and bind to cancer-related biomolecules. These targeting ligands can direct nanoparticles to specific tumor cells by attaching to the surface of the tumor. Polymers and surfactants with specific end terminal groups (hydroxyl, carboxyl and amine) can be tethered on the surface of nanoparticles and have been exploited for targeting ligands attachment [11, 12]. The highly efficient approach is based on covalent bonding between ligands and nanoparticles. One example is the conjugation of cetuximab, a chimeric antibody against cell surface epidermal growth factor receptor, through a thioether bond to the surface of poly-lactide-co-glycolideacid (PLGA) nanoparticles [13]. Such nanoparticles can be loaded with cytotoxic agents for targeted delivery. In this case, we plan to use ligands of HDACs to decorate the PLGA nanoparticle for intracellular targeted delivery. What is also unique in this study is the targeting of an intracellular component, HDACs, instead of a cell surface biomarker.

**Figure 1:**
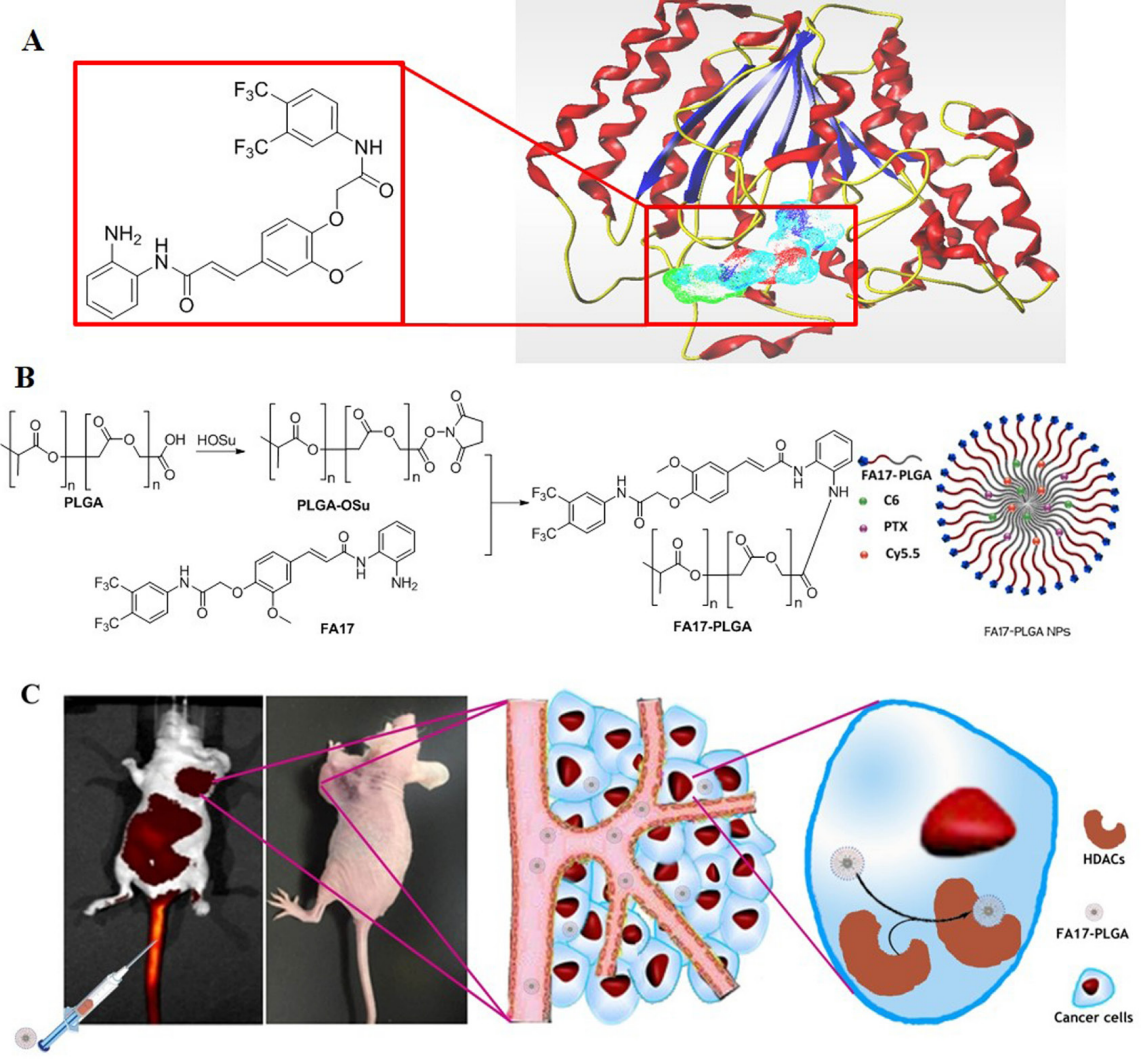
A schematic illustration of the FA17-PLGA NPs for intracellular delivery (**A**) Structure of FA17 and the complex of FA17 with HDAC. (**B**) Synthetic scheme of FA17-PLGA and schematic design of FA17-PLGA NPs. (**C**) Antitumor mechanism of FA17-PLGA NPs.

The search for intracellular drug delivery system that could efficiently deliver drugs to targeted cells and enhance their therapeutic effects is a very active field [14, 15]. Specifically, we developed a chemical modified polymer for the preparation of NPs. FA17 was developed in our previous study which can specifically interact with HDACs [16]. To optimize the intracellular delivery efficiency of the NPs, FA17 was chemically conjugated to PLGA (Figure 1). The cellular uptake and exocytosis of the actively targeting nanoparticles were investigated in MCF-7 cells with overexpression of HDACs. Moreover, the distribution and antitumor efficacy *in vivo* were investigated in xenograft mouse models.

## RESULTS AND DISCUSSION

### Preparation and characterization of polymers and NPs

For the construction of the proposed chemically modified NPs, we firstly prepared a new polymeric backbone through conjugation of FA17 at the end of the PLGA polymer (Supplementary Figures 1–3). Subsequently, NPs were prepared using PLGA (PLGA NPs) and FA17-PLGA (FPLGA NPs) by the oil-in-water emulsion solvent evaporation method. There were no significant differences in the encapsulation efficiency (EE) and the loading capacity (LC) between PLGA NPs and FPLGA NPs (Supplementary Table 1). Moreover, the characteristic parameters of the two NPs were similar. It can be inferred that conjugation of FA17 with PLGA did not significantly influence the characteristics of NPs. Polyvinyl alcohol (PVA) was used as a stabilizer for the emulsion preparation in virtue of its hydrophilic structure with abundant hydroxyl functional groups [17]. PVA could effectively improve the thermal stability and reduce the aggregation of NPs [18]. The slow release and high EE of coumarin-6 (C6)-PLGA NPs and C6-FPLGA NPs are consistent with previous reports [19] suggesting that C6 can be used to study the cellular uptake of NPs without stability problems. The release profiles of Paclitaxel (PTX) from PLGA NPs and FPLGA NPs were similar. The spatial distribution of the drug within the NPs depends on the polarity of the drug. Drugs that distribute to peripheral locations are more likely to be released [20]. The polarity of PTX is higher than that of C6, which explains the slow release of the latter from NPs. Transmission electron micrographs of the NPs in dispersion solution are presented in Supplementary Figure 4. All nanoparticles primarily existed as discrete, spherical or quasi-spherical forms with a smooth surface and narrow particle size distribution.

### Investigation of cellular uptake and NPs retention

**Figure 2:**
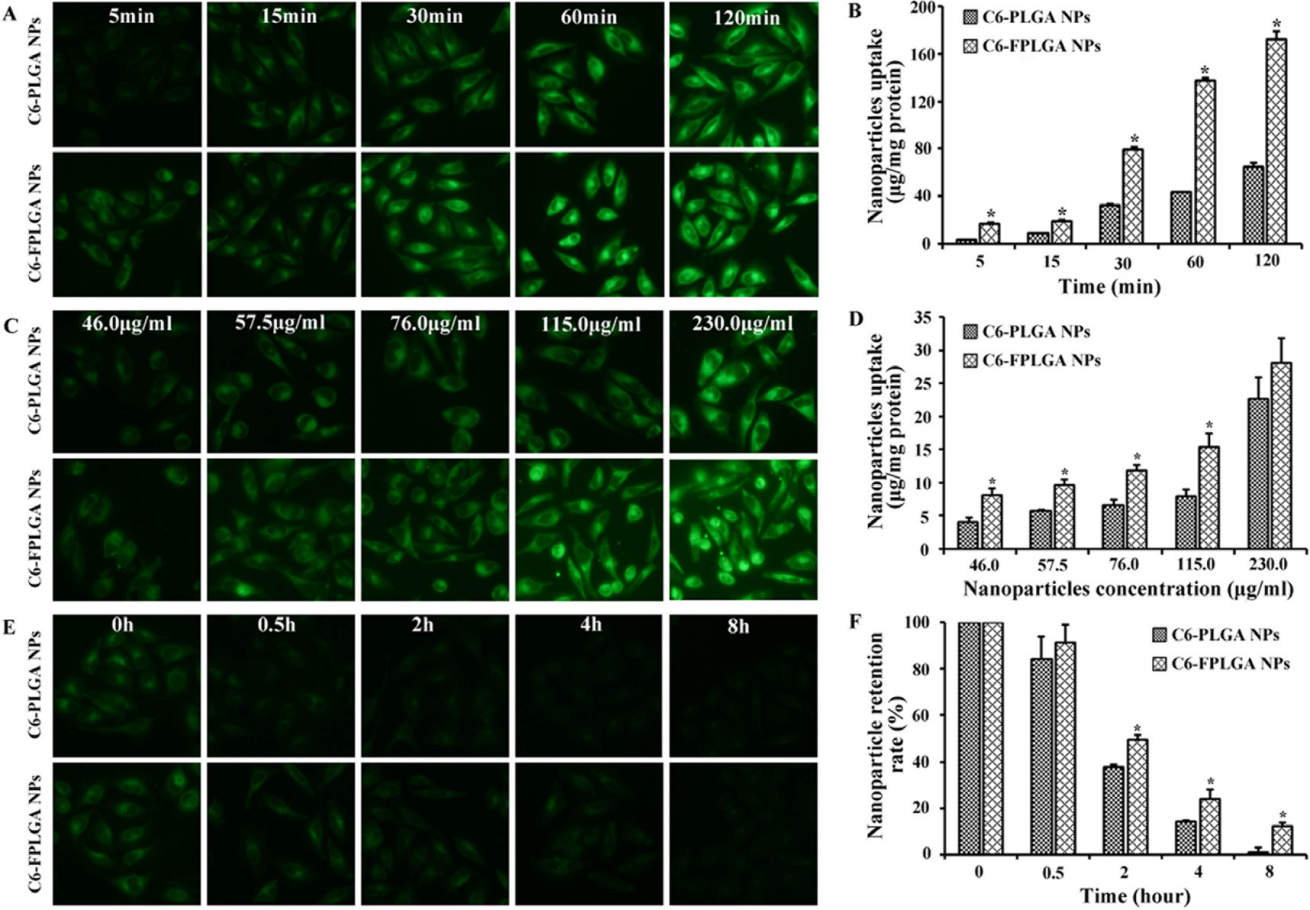
Cellular uptake and exocytosis of C6-PLGA NPs and C6-FPLGA NPs in MCF-7 cells Fluorescent microscope images (**A**) and quantitative analysis (**B**) of the time dependent cellular uptake. Fluorescent microscope images (**C**) and quantitative analysis (**D**) of the concentration dependent cellular uptake. Fluorescent microscope images (**E**) and quantitative analysis (**F**) of cellular exocytosis. Each value represents the mean ± standard deviation (*n* = 3). *P < 0.05 C6-FPLGA NP vs C6-PLGA NP.

We investigated the cellular uptake signatures of NPs in MCF7 cell line with overexpression of HDACs. It has been reported that HDAC1, 3 and 6 are the most highly expressed HDACs in MCF-7 [21]. The fluorescence images were recorded to visualize cellular uptake and NPs retention in MCF7 cells. Stronger green fluorescence in the cell was observed in C6-FPLGA NPs treated MCF-7 cells than that of C6-PLGA NPs. The proportion of MCF-7 cells with green fluorescence treated with both NPs for 120 min was much higher than that treated for 15 min. In contrast to C6-FPLGA NPs, only a small number of cells with fluorescence were observed in the C6-PLGA NP-treated group. Furthermore, the fluorescence intensity in MCF-7 cells treated with C6-PLGA NPs and C6-FPLGA NPs increased with the NPs concentration from 46 to 230 μg/mL (Figure 2C). The amount of C6-PLGA NPs and C6-FPLGA NPs internalized by cells was quantified. The internalized amount of C6-FPLGA NPs was 2.3–3.3 times higher than that of C6-PLGA NPs at 15, 30, 60 and 120 min (*P* < 0.05) (Figure 2B). There were significantly less internalization of C6-PLGA NPs than that of C6-FPLGA NPs in the low concentration range (*P* < 0.05). The cell uptake amounts of C6-FPLGA NPs at concentration of 46.0, 57.5, 76.0 and 115.0 μg/mL were 8.1, 9.7, 11.8 and 15.4 μg/mg protein, while the cellular uptake amounts of C6-PLGA NPs at corresponding concentration were 4.2, 5.7, 6.7 and 8.0 μg/mg protein (Figure 2D). After washing of the cells to remove the unbound NPs, the remaining NPs were quantitatively analyzed by measuring the fluorescent intensity. As shown in Figure 2E, the green fluorescence intensity of the cells showed time-dependent decreases. The retention percentages for C6-FPLGA NPs were 91%, 49% and 24% at 0.5, 2 and 4 h, respectively. In comparison, retention of C6-PLGA NPs were 84%, 37% and 13%, respectively (Figure 2F). The minimum intensity was observed at 8 h and the retention rate for C6-PLGA NPs was only 1.0%. The retention rate of C6-FPLGA NPs in cells was significantly higher than that of C6-PLGA NPs in the 2–8 h period (*P* < 0.05).

These results suggest that both C6-PLGA NPs and C6-FPLGA NPs were able to internalize into MCF7 cells in a time-dependent manner. However, the amount of intracellular NPs was similar at high concentrations, presumably due to the saturated binding capacity of the cell [22].

It is important to note that the level of cellular internalization of C6-FPLGA NPs was significantly higher than that of C6-PLGA NPs at low concentrations (46.0～115.0 μg/mL), suggesting that FPLGA could efficiently promote NPs taken by cell. Likewise, the results of NPs retention experiments indicated that FPLGA modification can delay the exclusion of C6-FPLGA NPs from cells and balanced with uptake of NPs [23]. The uptake of C6-FPLGA NPs occurs more quickly than that of C6-PLGA NPs. These results suggested that the modification of PLGA with FA17 was beneficial for the internalization and active targeting ability of NPs [24].

### Enhanced tumor accumulation of FPLGA NPs *in vivo*

**Figure 3:**
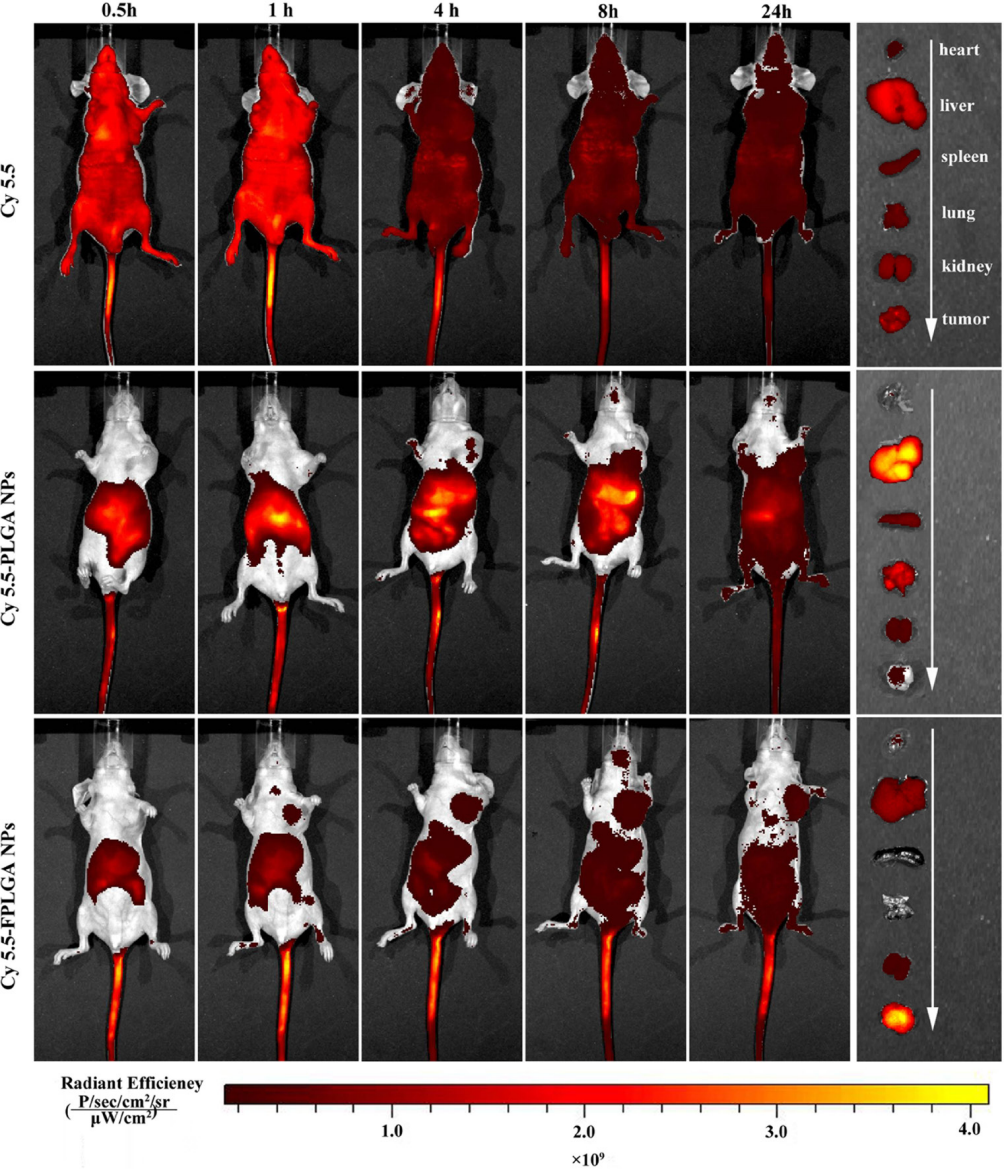
*In vivo* imaging of tumor bearing nude mice at different time after intravenous injection via the tail vein with free Cy5.5, Cy5.5-PLGA NPs and Cy5.5-FPLGA NPs

The biodistribution of NPs was investigated to evaluate its *in vivo* targeting efficiency as well as potential tissue toxicity. Non-invasive imaging was used to monitor the real-time distribution of free Cy5.5, Cy5.5-PLGA NPs and Cy5.5-FPLGA NPs in tumor-bearing mice. Figure 3 showed the *in vivo* near-infrared fluorescence images of mice at different time points after injection. After 0.5 h, a noticeable fluorescence signal of Cy5.5 was observed in entire body. At 4 h post-injection, although the fluorescence intensity of Cy5.5 was decreased, their distribution remained in the whole body throughout the experiment. The heart, liver, spleen, lung, kidney and tumor tissues of the mice were removed after 24 h. Similar fluorescence intensity was observed in every tissue except in the liver tissue where the fluorescence was slightly bright. These results suggested that the free Cy5.5 distribution was not selective for any organ or tissue in mice.

Cy5.5-PLGA NPs and Cy5.5-FPLGA NPs exhibited different distributions in tumor-bearing mice from 0.5 to 24 h post-injection (Figure 3). A higher fluorescence intensity of Cy5.5-PLGA NPs was observed in the abdominal cavity when compared to the Cy5.5-FPLGA NPs. The intensity gradually increased over from 0.5 to 8 h. At 24 h post-injection, the distribution area in abdominal cavity of mice treated with Cy5.5-PLGA NPs became much larger, but the intensity became weaker. It was noteworthy that the tumor targeting efficiencies of Cy5.5-PLGA NPs and Cy5.5-FPLGA NPs in mice were distinct. The fluorescence signal in the tumor site was observed at 1 h for Cy5.5-FPLGA NPs and at 4 h for Cy5.5-PLGA NPs, respectively. As time progressed, the fluorescence signal of Cy5.5-FPLGA NPs became increasingly distributed in the tumor tissue, eventually reaching its maximum at 8 h. At all the time points, there was a greater accumulation of Cy5.5-FPLGA NPs in the tumor when compared to Cy5.5-PLGA NPs. The distribution of Cy5.5-PLGA NPs and Cy5.5-FPLGA NPs in tissues was also assessed. For Cy5.5-PLGA NPs, the fluorescence signal was observed in liver, spleen, lung, kidney and tumor tissues. The fluorescent intensity of the tumor tissue was the lowest among all tissues examined. The fluorescence signal of Cy5.5-FPLGA NPs was found in liver, kidney and tumor tissues. The intensity of the tumor tissue was the highest among all examined tissues.

It is well known that the distribution of NPs after intravenous injection *in vivo* is influenced by many factors including the physiochemical properties of the NPs (e.g., size, shape chemistry surface and charge) [25]. The liver is a major organ in the reticuloendothelial system and is typically associated with NPs intake [26]. Therefore, it is reasonable to see that the intensity in the liver tissue for Cy5.5-PLGA NPs was the highest. The distribution of Cy5.5-FPLGA NPs *in vivo* was obviously different from that of Cy5.5-PLGA NPs. The only difference of these two NPs is that Cy5.5-FPLGA NPs was chemical modification with FA17. These results indicated that NPs modified with FA17 reduced retention in the liver, decreased drug distribution in the liver, spleen and lung, and increased concentration in tumor, showing good selective distribution *in vivo* [27–29]. It is feasible that PLGA NPs could work as an efficient drug delivery system and reduced systemic toxicity while improving drug delivery efficiency in cancerous tissue.

### Detection of antitumor efficacy and potential toxicity

**Figure 4:**
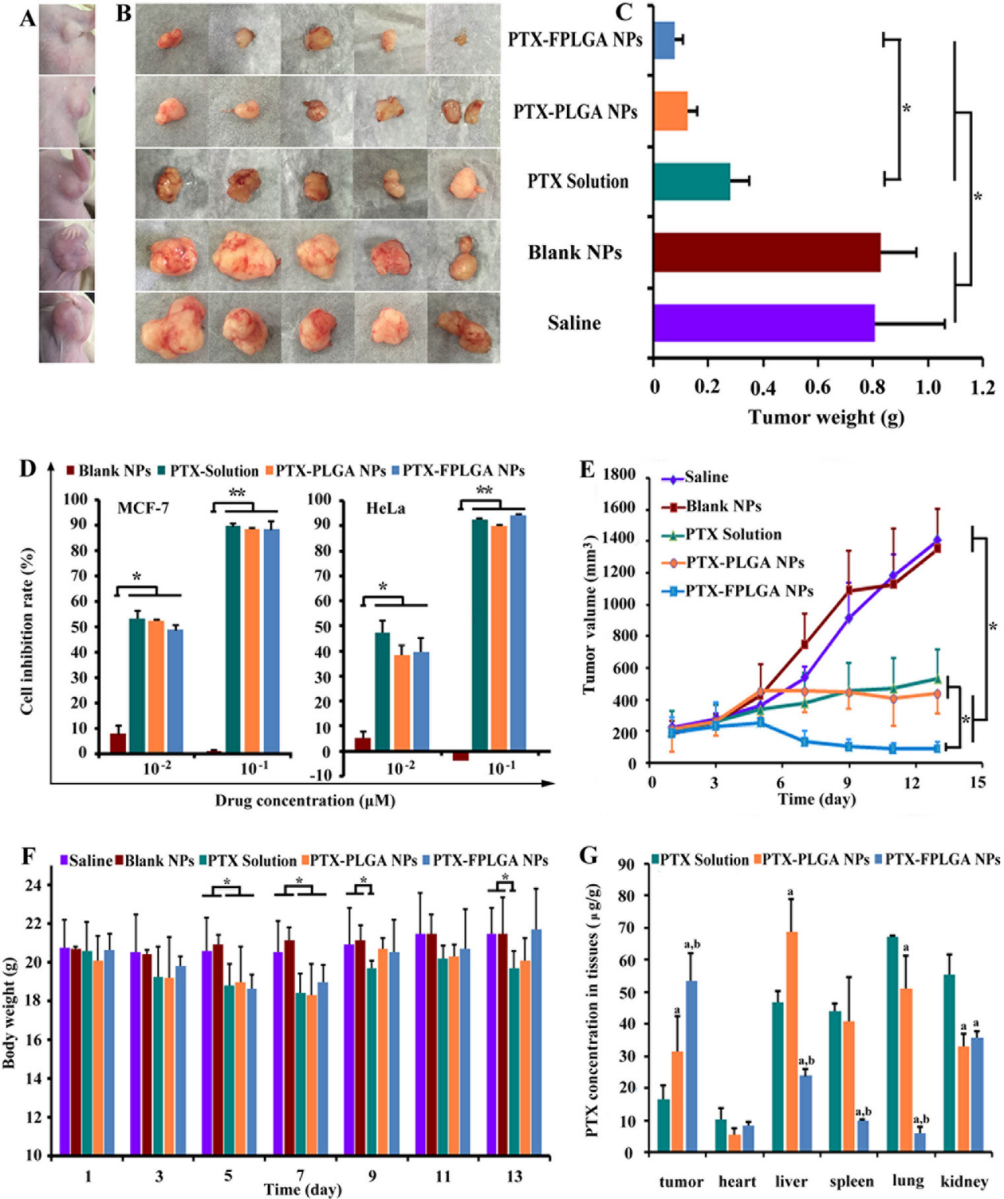
*In vivo* and *in vitro* anti-tumor capacities of PTX-PLGA NPs and PTX-FPLGA NPs Typical photographs showing the size of tumor in mice (**A**) and tumor collected from mice (**B**) on 13 days after five different treatments. Weight of the tumors collected from mice on 13 days (**C**). Inhibition rate in MCF-7 cells after five different treatments (**D**). Tumor growth curves of five different treatments groups (**E**). Body weight of the mice of the five different treatments groups (**F**). PTX concentration in different tissues after treated various drug formulations (**G**). *P < 0.05, **P < 0.01.

Then the *in vivo* antitumor efficacy was further investigated. Mouse body weight was recorded to evaluate the potential toxicity. Figure 4A and 4B show a photograph of the original tumor, which reflects the tumor inhibition efficacy on day 13 after the injection of saline, blank NPs, PTX solution, PTX-PLGA NPs and PTX-FPLGA NPs. The tumor volumes were significantly increased in the saline group and unmodified NPs group, going from 223 to 1408 mm^3^ and from 186 to 1352 mm^3^, respectively (Figure 4E). Treatment with PTX solution, PTX-PLGA NPs and PTX-FPLGA NPs exhibited potent antitumor activity. Tumor volumes in PTX-FPLGA NPs were significantly less compared to the saline and unmodified NPs groups (*P* < 0.05). Remarkably, the PTX-FPLGA NPs exhibited the highest antitumor ability and gradually reduced the tumor volume. The tumor volumes of the PTX-FPLGA NPs group were statistically different from the PTX solution and PTX-PLGA NPs groups (*P* < 0.05), while the difference between the latter two groups was not significant (*P* > 0.05). The differences in tumor weight among the groups (Figure 4C) showed a similar trend. The tumor inhibition rates were 90% for the PTX-FPLGA NPs group, 84% for the PTX-PLGA NPs group and 65% for the PTX solution group.

*In vitro* cytotoxicity studies were carried out on MCF-7 and HeLa cells, on which FA17 exhibited potent anti-proliferative activity. PTX solution, PTX-PLGA NPs and PTX-FPLGA NPs showed similar inhibition effects (*P* < 0.05), whereas the blank NPs alone did not inhibit cell growth (Figure 4D). It has been confirmed that loading PTX into NPs did not significantly influence its cytotoxic effects. The body weights of mice are shown in Figure 4F. PTX solution exhibited significant weight loss compared to the saline and blank NPs groups (*P* < 0.05). The body weight of mice treated with PTX-FPLGA NPs were somewhat higher than that of PTX-PLGA NPs, which suggests that the former displayed lower cytotoxic. Moreover, significantly more drug accumulated in the lung and kidney tissues of mice treated with PTX solution compared to those treated with PTX-PLGA NPs and PTX-FPLGA NPs (*P* < 0.05) (Figure 4G). Significantly higher levels of PTX were measured in the spleen tissue of the PTX solution and PTX-PLGA NPs groups. The PTX concentration in liver tissue of the PTX-PLGA NPs group reached 68.8 μg/g, which was significantly higher than that of the PTX solution and PTX-FPLGA NPs groups (*P* < 0.05). In particular, the extremely high accumulations in the tumor tissue were detected in the PTX-FPLGA NPs group with concentration increased 1.7-fold than that of PTX-PLGA NPs group and 3.3-fold than that of PTX solution group (*P* < 0.05).

These results suggest that FPLGA NPs exhibited improved antitumor potency and reduced potential cytotoxic compared to PLGA NPs or chemotherapy alone. Quantitative analyses of the three formulations of PTX in different tissues indicated that the encapsulation of drugs in FPLGA NPs could give rise to an increased accumulation of drugs in the tumor tissues [30, 31]. Moreover, combining with the results of *in vivo* distribution, we can obtain FPLGA NPs preferentially accumulated in tumor tissues due to the EPR effect and the active target-ability of FA17 [32]. The results of improved antitumor efficacy of FPLGA NPs were also consistent with its enhanced cellular uptake and longer retention time in MCF cells [33]. The side-effects were assessed by recording body weight changes and mortality, which suggested that modified NPs could reduce the undesired toxicity [34].

## MATERIALS AND METHODS

(*2E*)-*N*-(2-Aminophenyl)-3-[4-(2-{[3,5-bis(trifluoro-methyl)phenyl]amino}-2-oxoethoxy)-3-methoxyphenyl]acryl-amide (FA17) was designed and synthesized in our laboratory. The poly-lactide-co-glycolide acid (PLGA, copolymer ratio 50:50, molecular weight (MW) = 20,000 Da) was a friendly gift from Purac Biochem BV. (Gorinchem, Netherlands). Polyvinyl alcohol (PVA, average MW 72, 600–81,400) was purchased from Shanxi Sanwei Group Co., Ltd. (Shanxi, China). *N*-Ethyl-*N’*-(3-dimethylaminopropyl) carbodiimide hydrochloride (EDCI) was purchased from Nanjing DuoDian Chemical Co., Ltd. (Jiangsu, China). *N*-hydroxysuccinimide (NHS) was obtained from Sinopharm Chemical Reagent Co., Ltd. (Shanghai, China). Paclitaxel (PTX) was purchased from Fujian Kerui Pharmaceutical Co., Ltd. (Fujian, China). Coumarin-6 was supplied by Heowns Biochemical Technology Co., Ltd. (Tianjin, China). CyDye mono-reactive NHS ester (Cy5.5) was obtained from Beijing Fanbo Biochemicals Co., Ltd. (Beijing, China). BCA Protein Assay Kit was purchased from BeiJing Cowin Biotech Co., Ltd. ^1^H-NMR spectra were recorded on a Bruker AVANCF 400 MHz spectrometer. UV/vis spectra were measured on a UV1600 spectrophotometer (Zhengjiang Fuli Analytical Instrument Co., Ltd.). FT-IR spectra were recorded on a Shimadzu 8400S infrared spectrophotometer (Shimadzu, Japan). Transmission electron microscopy (TEM) images were captured using a JEM-2100F (Electronics Corporation of Japan). Size analysis was performed on a laser scattering Particle Sizer (HYL-1080, DanDong Hylology Instruments Co., Ltd.). Fluorescence measurements were performed on a fluorescence spectrophotometer (RF-5301pc, SHIMADZU). Fluorescence images were captured using a fluorescence microscope (Nikon camera) and *in vivo* images were captured using a Xenogen IVIS Spectrum (Xenogen).

### Preparation of PLGA and FA17-PLGA nanoparticles

Nanoparticles were prepared using PLGA and FA17-PLGA by the oil-in-water emulsion solvent evaporation method. PTX (10 mg) was dissolved in 2 mL dichloromethane containing 60 mg of PLGA or FA17-PLGA. Next, the solution was injected drop-wise into an aqueous polyvinyl alcohol (PVA) solution (20 mL, 1% w/v) to form an oil-in-water emulsion. The emulsion was subjected to an ultrasonic cell crusher for 60 s at 400 W in an iced water bath and then stirred overnight on a magnetic stir plate at room temperature to evaporate the organic solvent. PTX loaded PLGA nanoparticles (PTX-PLGA NPs) or PTX loaded FA17-PLGA nanoparticles (PTX-FPLGA NPs) were collected by ultracentrifugation at 12,000 rpm, 4°C for 15 min and washed three times with doubly distilled water.

Coumarin-6 loaded PLGA and FA17-PLGA nanoparticles (C6-PLGA NPs, C6-FPLGA NPs) as well as Cy5.5 loaded PLGA and FA17-PLGA NPs (Cy5.5-PLGA NPs, Cy5.5-FPLGA NPs) were prepared as described above with one exception, the 10 mg of PTX was replaced with either 1.0 mg of coumarin-6 or 0.5 mg of Cy5.5.

### Biological evaluation of nanoparticles

### Time dependence of cellular uptake

MCF-7 cells were seeded in 6-well plates at a density of 2 × 10^5^ cells/well and incubated for 24 h. Either C6-PLGA NPs or C6-FPLGA NPs suspension (approximate 115.0 μg/mL) was added into the well followed by further incubation of 5, 15, 30, 60 and 120 min. The concentration of coumarin-6 in the two types of nanoparticles was 1.7 μg/mL. After the suspension was removed, the cells were washed three times with ice-cold PBS (0.01 M, pH 7.4) and imaged with a fluorescence microscope using green excitation filters. Subsequently, the cells were trypsinized, harvested and re-suspended in 3 mL of methanol. The cell was disrupted using an ultrasonic cell crusher and the supernatant was collected by centrifugation at 6,000 rpm for 10 min. The mean fluorescence intensity of the collected supernatant was measured using a fluorescence spectrophotometer operated at a λ_ex_ of 497 nm and a λ_em_ of 523 nm. The cell pellets were then washed with PBS and the total cell protein content in each well was determined using the BCA protein assay that accorded to the manufacturer's instructions. The nanoparticles amount in the cell was expressed as the amount of absorbed coumarin-6 (μg) per mg cell protein. All measurements were performed in triplicate.

### Concentration dependence of cellular uptake

MCF-7 cells were incubated for 24 h and then the culture medium was replaced with various concentrations of C6-PLGA NPs or C6-FPLGA NPs suspension (46.0, 57.5, 76.0 115.0 230.0 μg/mL). After 15 min of incubation, the cells were washed three times with ice-cold PBS and imaged with a fluorescence microscope. Subsequent steps were the same as described in section “Time dependence of cellular uptake”.

### Nanoparticles retention in cells

MCF-7 cells were seeded in 6-well plates and incubated for 24 h. C6-PLGA NPs or C6-FPLGA NPs suspension (115.0 μg/mL) was added into the well and incubated for 15 min. The cells were washed three times with PBS to remove unbound nanoparticles and then incubated again with DMEM for 0, 0.5, 2, 4, 8 h, respectively. Subsequently, the cells were washed three times with ice-cold PBS and imaged with a fluorescence microscope. Subsequent steps were the same as described in section “Time dependence of cellular uptake”. The nanoparticles retention rate (%) in cell was calculated using the following equation:

$$\text{Nanoparticles retention rate}\left(\%\right)=\frac{\text{Amount of nanoparticles in cells at t h}}{\text{Amount of nanoparticles in cells at }0\text{ h}}\times 100$$

### *In vitro* cytotoxicity assay

MCF-7 and HeLa cells were seeded at a density of 1×10^4^ cells per well in a 96-well plate and allowed to adhere for 24 h. The medium was then replaced with fresh medium containing different concentrations of paclitaxel, PTX-PLGA NPs or PTX-FPLGA NPs (0.01 and 0.1 μM paclitaxel). Blank nanoparticles of the same PLGA content were used as controls. After 48 h of incubation, 20 μL of 5 mg/mL MTT solution was added to each well and cultured for 4 h. Next, the MTT medium was discarded and dimethylsulfoxide (100 μL/well) was added. The absorbance of the DMSO solution was measured at a wavelength of 490 nm using an ELISA plate reader. The cell inhibition rates after treatment with the parent drug and nanoparticles were assayed according to the MTT method.

### *In vivo* evaluation of nanoparticles

### Animal tumor model

To establish breast tumor-bearing mice, 200 μL of a suspension containing MCF-7 cells (5 × 10^6^ cells/mL) were injected subcutaneously into the leftlimb armpits of Nude female Bala/c-numice. The *in vivo* imaging experiments were carried out in mice with a tumor volume of approximately 500 mm^3^. The anti-tumor efficacy experiments were carried out in mice with tumor volumes of approximately 200 mm^3^. The tumor volume (V) was calculated as: V = (L × S^2^)/2, where L is long diameter and S is short diameter determined by vernier caliper. The tumor inhibition rate (IR) was calculated as: IR% = (Wc-W)/Wc × 100, where Wc is the tumor weight of the control group and W is the tumor weight of treatment group.

### *In vivo* imaging

Tumor-bearing mice were separately injected with Cy5.5 solution, Cy5.5-PLGA NPs and Cy5.5-FPLGA NPs via the tail vein. The Cy5.5 concentration (0.01 nmol/g body weight) was equivalent across all treatments. At 0.5, 1, 4, 8 and 24 h post-injection, the mice were anesthetized with isoflurane and fluorescent images were acquired at an excitation wavelength of 675 nm and an emission wavelength of 720 nm using a Xenogen IVIS Spectrum. After 24 h, mice were sacrificed and the tissues of interest were collected for further Xenogen IVIS Spectrum analysis.

### *In vivo* anti-cancer efficacy

Once the tumor volume reached approximately 200 mm^3^, the mice were randomly divided into five groups (*n* = 5 for each group): 1) control group (sterile isotonic saline); 2) blank nanoparticles group (8.5 mg/mL nanoparticles without PTX); 3) PTX solution group;4) PTX-PLGA NPs group; and 5) PTX-FPLGA NPs group. The concentration of PTX (1 mg/mL) was the same across all treatment groups (i.e., PTX solution, PTX-PLGA NPs and PTX-FPLGA NPs). All groups received a 20 mL/kg body weight dose via intraperitoneal injection on days 1, 3, 5, 7, 9, 11, and 13. Tumor size and body weight were assessed every other day. Tumor volume-time curves were drawn to compare the anti-tumor efficacy across the different PTX formulations. The mice were euthanized by cervical dislocation on day 13 after the first injection. The tissues (tumor, heart, liver, spleen, lung and kidney) were collected, wiped with filter paper and weighed. The tissues were homogenized in isotonic saline and PTX was extracted using diethyl ether. The amount of PTX in the tissue was determined using HPLC as described previously.

### Statistical analysis

Results were expressed as the mean ± standard deviation (SD). Statistical comparisons were made using the ANOVA. *P* < 0.05 was considered to be statistically significant.

## CONCLUSIONS

In summary, we have developed functionalized NPs through conjugation of FA17 with PLGA. Due to the affinity between FA17 and HDACs, these functionalized NPs showed significantly enhanced internalization into MCF-7 cells compared to PLGA NPs, and such functionalization did not influence drug load or release. *In vivo* experiments revealed that FPLGA NPs delivered PTX to the tumor site more efficiently than PLGA NPs and consequently exhibit potent antitumor effects *in vivo*. Therefore, the chemically modification endowed the functionalized intracellular drug delivery system with active targeting ability to cancer cells. Enhanced drug distribution in tumor tissue and potent antitumor efficacy were observed *in vivo*. In light of these results, such HDAC-targeting NPs could be a promising intracellular drug delivery system.

## SUPPLEMENTARY MATERIALS FIGURES AND TABLES



## Abbreviations

C6: Coumarin-6
NPs: nanoparticles
EPR: enhanced permeability and retention
PLGA: poly-lactide-co-glycolideacid
EE: encapsulation efficiency
LC: loading capacity
PVA: polyvinyl alcohol
PTX: paclitaxel
