# Supplementary material for: Homing in on an intracellular target for delivery of loaded nanoparticles functionalized with a histone deacetylase inhibitor
Source: Oncotarget. 2017 Aug 7;8(40):68242–51. doi: 10.18632/oncotarget.20021 (PMC5620252; doi:10.18632/oncotarget.20021)
Supplement: Supplementary file 1 [file oncotarget-08-68242-s001.pdf]

Homing in on an intracellular target for delivery of loaded nanoparticles functionalized with a histone deacetylase inhibitor

SUPPLEMENTARY MATERIALS

Synthesis and characterization of FA17-PLGA conjugates

PLGA (1.0 g, 25 μmol) and NHS (12 mg, 100 μmol) were dissolved in 40 mL of anhydrous dichloromethane at room temperature. Subsequently, the mixture was cooled to 0°C and 2.5 mL of triethylamine and EDCI (20 mg, 100 μmol) were added slowly. The reaction mixture was stirred at room temperature for another 10 h to afford the activated ester, PLGA-NHS. The HDAC inhibitor FA17 (138 mg, 250 μmol) in 4 mL of anhydrous dichloromethane was added to the reaction mixture. After stirring for 18 h at room temperature, the mixture was diluted with 4 mL of dichloromethane. The organic layer was washed with saturated $NaHCO_3$ solution, brine, and 2 mol/L HCl, and dried over anhydrous Na_2SO_4. The desired product (FA17-PLGA) was obtained by filtration and concentration *in vacuo* and characterized using ^{1}H-NMR, FTIR and UV/vis spectroscopy. As shown in Supplementary Figure 1, the presence of major vibrational features associated with PLGA and FA17 can be observed in the spectra. PLGA displays a prominent peak at 1759 cm^{-1} corresponding to the stretching of the –C = O bond of the polymer. The broad peak at 3294 cm^{-1} is due to N-H stretching and the peak at 748 cm^{-1} is the result of bending vibrations of the benzene ring. After the conjugation of FA17 at the end of the PLGA polymer, the presence of two salient peaks at 1620 cm^{-1} and 1504 cm^{-1} can be attributed to the newly formed amide functional group. They are two characteristic bands corresponding to C=O and CN-H vibrations, respectively. Such results indicate the formation of the amide bond.

The ^{1}H-NMR spectrum of FA17-PLGA was used to further characterize the structure of the conjugate (Supplementary Figure 2). The characteristic signals appearing between 6.90 ppm and 7.50 ppm were assigned to the aromatic ring of FA17. The peaks at 5.19–5.32, 4.70–4.80, and 1.33-1.60 ppm were assigned to the methine hydrogen of the lactide units, methylene hydrogen of the glycolide units and the methyl hydrogen of the lactide units, respectively. The peak at 7.28 ppm was attributed to the signal of residual $CHCl_3$ from $CDCl_3$.

There was no significant absorbance of PLGA in the 200 to 400 nm range. The optical absorbance of the HDAC inhibitor FA17 and the modified polymer FA17-PLGA ranged from 250 nm to 300 nm (Supplementary Figure 3). In summary, all the above results confirm the successful conjugation of FA17 to PLGA.

Characterization of nanoparticles

Particle size and morphology

The size and size distribution of nanoparticles were determined using a laser scattering Particle Sizer with the concentration being 0.5 mg/mL. Transmission electron micrographs of the six types of nanoparticles in dispersion solution. The average particle size of the six nanoparticle types (i.e., C6-PLGA NPs, C6-FPLGA NPs, PTX-PLGA NPs, PTX-FPLGA NPs, Cy5.5-PLGA NPs and Cy5.5-FPLGA NPs) changed from 365 nm to 465 nm (Supplementary Table 1). The span of these nanoparticles changed from 0.448 to 0.564, which indicated monodisperse nanoparticles dispersion.

Transmission electron micrographs of the six types of nanoparticles in dispersion solution are presented in Supplementary Figure 4. All nanoparticles primarily existed as discrete, spherical or quasi-spherical forms with a smooth surface and narrow particle size distribution.

Drug encapsulation efficiency and loading capacity

The drug encapsulation efficiency (EE) and loading capacity (LC) of PTX-PLGA NPs and PTX-FPLGA NPs were determined by a HPLC system as previously described. Paclitaxel-loaded nanoparticles (0.5 mg) were dispersed in 1mL of methanol and shaken for 30 min. The samples were centrifuged at 12000×g for 10 min at 25°C, and the supernatant was collected for determination. The contents of coumarin-6 and Cy5.5 loaded nanoparticles were determined by a fluorescence spectrophotometer. Briefly, 1.0 mg fluorescein-loaded nanoparticles were dispersed in 5 mL of methanol and treated using the same process as mentioned above. Coumarin-6 had an excitation wavelength (λ_{ex}) of 497 nm and an emission wavelength (λ_{em}) of 523 nm. Cy5.5 had an λ_{ex} of 673 nm and an λ_{em} of 692 nm. The standard curve was established by performing a linear regression between the concentration and the intensity.

All analyses were performed in triplicate. EE and LC were calculated using the following formulae:

$$EE\ (\%) = (Qt\text{-}Qm)/Qt\times100$$
$$LC\ (\%) = (Qt\text{-}Qm)/Qp\times100$$

Where Qp is the total amount of material, Qt is the amount of drug in formulation and Qm is the drug content in the supernatant.

***In vitro* drug release**

The release behavior of paclitaxil and coumarin-6 from the nanoparticles was evaluated in PBS (pH 7.4) containing 0.1% v/v of Tween 80. Paclitaxel or coumarin-6 loaded nanoparticles were dispersed in 2.0 mL of PBS and placed in a dialysis bag (MWCO 8000 Da). Then dialysis bag was immersed into another 20.0 mL of PBS at 37°C and shaken at a constant speed of 100 r/min. At appropriate intervals, 5 mL of the release medium were withdrawn and an equal volume of fresh PBS was added. The paclitaxil and coumarin-6 amounts were determined by HPLC and fluorescence spectrophotomete.

The encapsulation efficiency (EE) of PLGA NPs and FPLGA NPs ranged from 65% to 77% after loading with PTX and Cy5.5 (Supplementary Table 1). There were no significant differences in the EE between nanoparticles prepared with either PLGA or FPLGA. It is noted that the EE of coumarin-6 loaded nanoparticles (including C6-PLGA NPs and C6-FPLGA NPs) was very high, and their EE were 98% and 89%, respectively. Likewise, the loading capacity (LC) is similar for PLGA NPs and FPLGA NPs after loading each model drug. In order to improve the LC of PTX inside PLGA NPs and FPLGA NPs, the dosage of PTX in formulation was increased, which was 10 times and 20 times higher than those of C6 and Cy5.5, respectively. So the LC for PTX-PLGA NPs was 10.8% and for PTX-FPLGA NPs was 12.8%, respectively. The cumulative release rate was all less than 6% for C6-PLGA NPs and C6-FPLGA NPs after 14 days of *in vitro* release experiment. The data indicated the release of C6 from NPs was very slow. The slow release and high EE of C6-PLGA NPs and C6-FPLGA NPs are consistent with previous reports suggesting that C6 can be used to study the cellular uptake of NPs without stability problems. The release profiles of PTX from PLGA NPs and FPLGA NPs were also similar. On the first day, the cumulative release rate was 10.3% for PTX-PLGA NPs and 12.1% for PTX-FPLGA NPs. By the fourteenth day, the PTX percentages released from PTX-PLGA NPs and PTX-FPLGA NPs reached to 56.4% and 67.8%, respectively. Generally, the spatial distribution of the drug within the NPs depends on the polarity of the drug. Hydrophobic drugs distribute into the core or skeleton of NPs, while drugs with intermediate or high polarity occupy more peripheral positions. Drugs that distribute to more peripheral locations are more likely to be released. The molecular polarity of PTX is higher than that of C6, which explains the slow release of the latter from NPs.

Supplementary Table 1: Characteristics of different nanoparticles (mean ± S.D. *n* = 3)

Nanoparticles	Particle Size (nm)	Span	EE (%)	LC (%)
C6- PLGA NPs	375 ± 45	0.506	97.8 ± 1.2	1.63 ± 0.03
C6-FPLGA NPs	387 ± 37	0.544	88.8 ± 1.8	1.50 ± 0.02
Cy5.5-PLGA NPs	376 ± 26	0.497	73.6 ± 1.1	0.30 ± 0.01
Cy5.5-FPLGA NPs	365 ± 33	0.448	68.5 ± 0.8	0.30 ± 0.01
PTX-PLGA NPs	451 ± 41	0.564	65.4 ± 0.9	10.8 ± 0.3
PTX-FPLGA NPs	465 ± 55	0.544	77.3 ± 1.1	12.8 ± 0.2

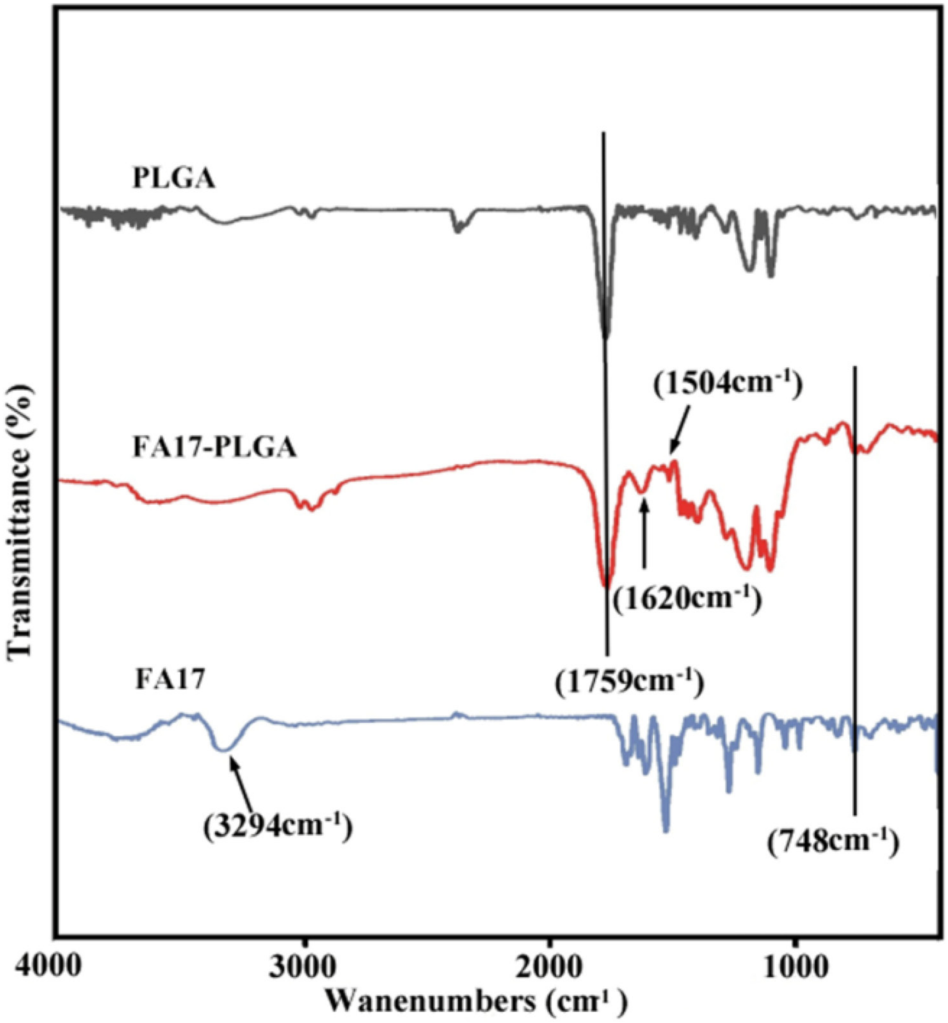

Supplementary Figure 1: FT-IR spectrum of PLGA, FA17-PLGA and FA17.

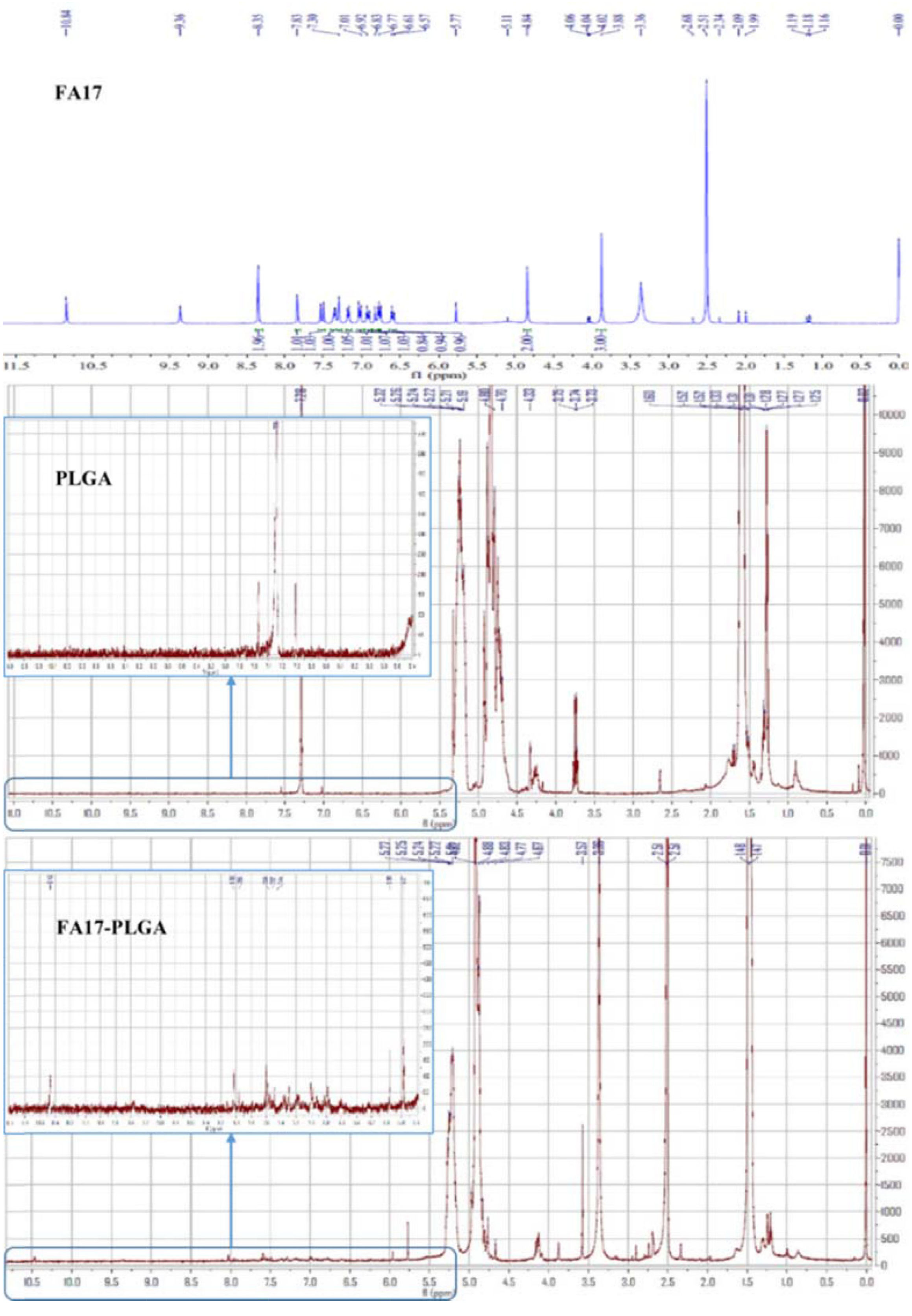

Supplementary Figure 2: ^{1}H NMR spectrum of FA17, PLGA and FA17-PLGA ($CDCl_3$, 400MHz).

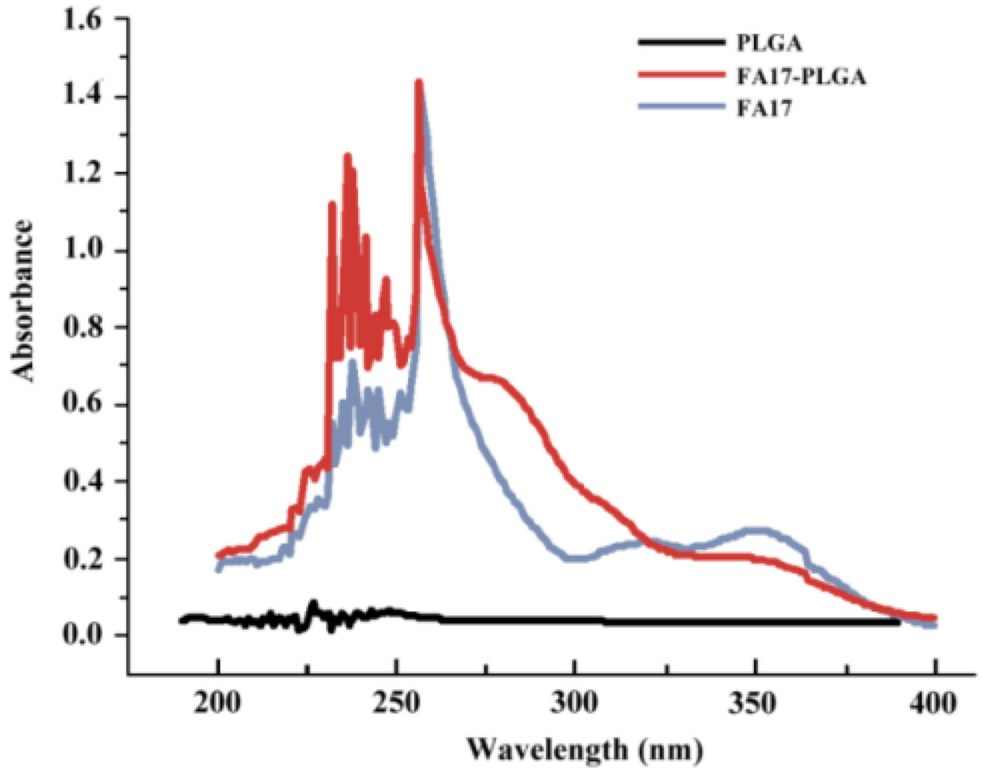

Supplementary Figure 3: UV-Vis spectrum of PLGA, FA17-PLGA and FA17.

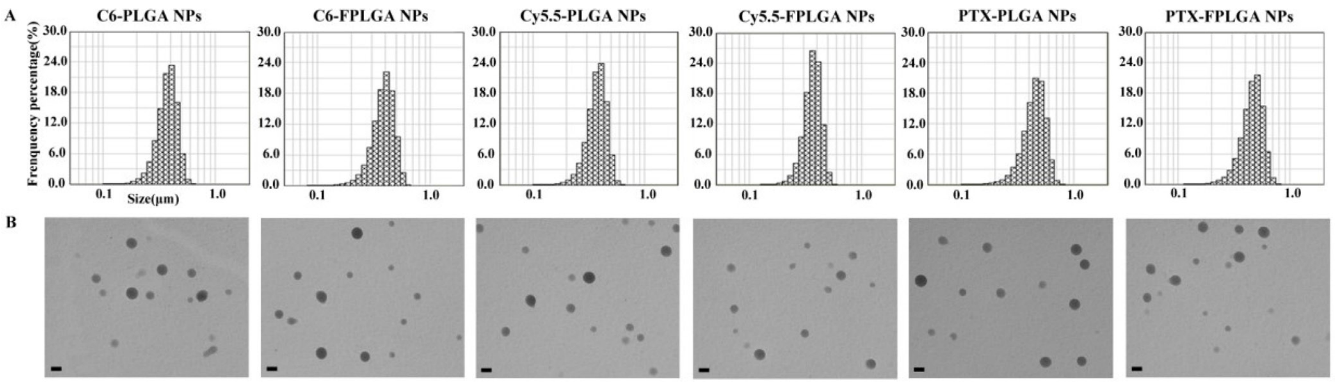

Supplementary Figure 4: Size distribution and morphology of nanoparticles. The size distribution of PLGA NPs and FPLGA NPs loading C6, Cy5.5 and PTX, respectively (**A**) and the TEM morphology of PLGA NPs and FPLGA NPs loading C6, Cy5.5 and PTX, respectively (**B**).Scale bar = 400 nm.
